# Hyperactivity of medial prefrontal cortex pyramidal neurons occurs in a mouse model of early-stage Alzheimer’s disease without *β*-amyloid accumulation

**DOI:** 10.3389/fphar.2023.1194869

**Published:** 2023-07-03

**Authors:** Nasreen Choudhury, Lihua Chen, Lena Al-Harthi, Xiu-Ti Hu

**Affiliations:** Department of Microbial Pathogens and Immunity, Rush University Medical Centre, Chicago, IL, United States

**Keywords:** Alzheimer’s disease, neurocognition, beta-amyloid, medial prefrontal cortex, pyramidal neuron, hyperactivity, neurotoxicity, ionotropic glutamate receptors

## Abstract

The normal function of the medial prefrontal cortex (mPFC) is essential for regulating neurocognition, but it is disrupted in the early stages of Alzheimer’s disease (AD) before the accumulation of Aβ and the appearance of symptoms. Despite this, little is known about how the functional activity of medial prefrontal cortex pyramidal neurons changes as Alzheimer’s disease progresses during aging. We used electrophysiological techniques (patch-clamping) to assess the functional activity of medial prefrontal cortex pyramidal neurons in the brain of 3xTg-Alzheimer’s disease mice modeling early-stage Alzheimer’s disease without Aβ accumulation. Our results indicate that firing rate and the frequency of spontaneous excitatory postsynaptic currents (sEPSCs) were significantly increased in medial prefrontal cortex neurons from young Alzheimer’s disease mice (4–5-month, equivalent of <30-year-old humans) compared to age-matched control mice. Blocking ionotropic glutamatergic NMDA receptors, which regulate neuronal excitability and Ca^2+^ homeostasis, abolished this neuronal hyperactivity. There were no changes in Ca^2+^ influx through the voltage-gated Ca^2+^ channels (VGCCs) or inhibitory postsynaptic activity in medial prefrontal cortex neurons from young Alzheimer’s disease mice compared to controls. Additionally, acute exposure to Aβ42 potentiated medial prefrontal cortex neuronal hyperactivity in young Alzheimer’s disease mice but had no effects on controls. These findings indicate that the hyperactivity of medial prefrontal cortex pyramidal neurons at early-stage Alzheimer’s disease is induced by an abnormal increase in presynaptic glutamate release and postsynaptic NMDA receptor activity, which initiates neuronal Ca^2+^ dyshomeostasis. Additionally, because accumulated Aβ forms unconventional but functional Ca^2+^ channels in medial prefrontal cortex neurons in the late stage of Alzheimer’s disease, our study also suggests an exacerbated Ca^2+^ dyshomeostasis in medial prefrontal cortex pyramidal neurons following overactivation of such VGCCs.

## 1 Introduction

The accumulation of *β*-Amyloid (Aβ) has long been considered a key hallmark of Alzheimer’s disease (AD) (LaFerla and Oddo, 2005; Querfurth and LaFerla, 2010) and linked to neuronal Ca^2+^ imbalance (Xie, 2004; Mattson, 2007; Small et al., 2009; Yu et al., 2009; Tong et al., 2018). However, its exact role in the development of AD is still debated (Hu, 2020; Zhang et al., 2021). Previous studies shows that Aβ levels increase in the brains of AD patients and decrease in the cerebrospinal fluid (CSF) of some early-stage AD patients before the formation of Aβ plaques and the onset of symptoms (Buckner et al., 2005; Palmqvist et al., 2017). Imaging studies using positron emission tomography (PET) and functional magnetic resonance imaging (fMRI) also suggest that Aβ release and its accumulation begin in specific brain regions, such as the prefrontal cortex (PFC) and hippocampus (HIPP) (Palmqvist et al., 2017); which both play a critical role in regulating neurocognition, before the appearance of Aβ plaques and neurocognitive decline (Maillet and Rajah, 2013; Sampath et al., 2017).

There is a lack of scientific knowledge regarding how AD alters the functional activity of live neurons in the PFC and HIPP in the brain, and whether Aβ causes such neuronal dysfunction during AD progression and aging. Previous studies suggest that Aβ not only impacts NMDA receptors (NMDARs) and voltage-gated L-type Ca^2+^ channels (L-channels) (Mattson, 2007; Hu, 2020), but also creates unconventional but functional Ca^2+^-permeable channels that have similar structure and function of the L-channel (Arispe et al., 1993; Pollard et al., 1993; Abramov et al., 2004; Lal et al., 2007; Shirwany et al., 2007; Itkin et al., 2011). These dysfunctions lead to neurotoxicity (Xie, 2004; Mattson, 2007; Small et al., 2009; Yu et al., 2009; Tong et al., 2018) in glutamatergic pyramidal neurons (Li et al., 2010; Kim and Rhim, 2011), which make up 80%–90% of neurons in the PFC and HIPP (Gabbott et al., 1997; Yuste, 2005). However, the extent to which Aβ affects the functional activity of live PFC neurons is still unclear, especially in the different stages of AD. The role of L-channel dysfunction and NMDAR overactivation (Xie, 2004; Mattson, 2007; Small et al., 2009; Yu et al., 2009; Tong et al., 2018), in causing intracellular Ca^2+^ dyshomeostasis, neurotoxicity, and therefore potentially contributing to AD neuropathology is also not well understood (Lal et al., 2007; Mattson, 2007; Shirwany et al., 2007; Itkin et al., 2011; Hu, 2016; Hu, 2020).

The earliest accumulation of Aβ in the brain occurs primarily in the orbital/medial PFC (o/mPFC) and HIPP, brain regions that are anatomically connected and functionally interplay to regulate neurocognition Buckner et al., 2005; Palmqvist et al., 2017). However, despite extensive research focusing on the neuropathology of the HIPP, the mPFC, another crucial regulator of neurocognition, is understudied, and therefore, is of particular interest to us (Hu, 2016; Hu, 2020).

Previous studies have found that *early* onset/familial AD gene mutations, such as presenilin 1/2 and amyloid precursor protein, can be detected in the carriers at very early-stage AD when cognitive abilities are still intact (Selkoe, 1997; Selkoe, 2001; Chhatwal et al., 2013; Van Cauwenberghe et al., 2016). In the carriers of *late* onset/sporadic AD gene mutation, such as apolipoprotein E (*APOE*4), dysfunction of neurons in the PFC/HIPP also occurs before fibrillar Aβ deposition and the clinical symptoms of AD (Persson et al., 2008; Sheline et al., 2010). However, it is still unclear how early-stage AD neuron dysfunction in the PFC and HIPP may contribute to the mechanism(s) by which Aβ enhances dysfunction of the HIPP and PFC in the later stages of this disease.

A previous study suggests that mPFC pyramidal neurons are responsible for the earliest release and deposition of Aβ in AD (Palmqvist et al., 2017). Vibrissal stimulation on one side also leads to an increase in Aβ levels in the contralateral barrel cortex (Bero et al., 2011). Aβ production and secretion are linked to synaptic activity (Cirrito et al., 2005; Cirrito et al., 2008), which is altered in the context of AD (Javonillo et al., 2022). Additionally, Aβ inhibits glutamate uptake (Lauderback et al., 1999) and disturbs extracellular potassium (K+) balance (Cheung et al., 2015; Price et al., 2021) by interfering with astrocyte activity (Beardsley and Hauser, 2014; Cheung et al., 2015; Kadala et al., 2015), leading to an aberrant increase in extracellular glutamate [(glut)o] and K+ [(K+)o] levels and promoting neuronal excitotoxicity in the PFC and HIPP.

Studies in animal models of AD, such as 3xTg-AD mice, suggest that the HIPP and PFC are among the earliest brain regions to experience Aβ release and deposition, starting at ∼6–12 months of age (6–12 months; equivalent of 30–43-year-old humans; see https://www.jax.org/news-and-insights/jax-blog/2017/november/when-are-mice-considered-old#) (LaFerla and Green, 2012; Puzzo et al., 2015), but not younger (Belfiore et al., 2019). However, there is a lack of research on pyramidal neuron dysfunction in these regions during early stages of AD (Ghatak et al., 2021a; Ghatak et al., 2021b; Javonillo et al., 2022).

Previous findings from early-stage AD study (Buckner et al., 2005; Palmqvist et al., 2017; Hu, 2020; Zhang et al., 2021) align with our perspective (Hu, 2020) but is conflicting with the Aβ theory. It therefore motivates us to advance our understanding of neuron dysfunction in the brain regions susceptible to Aβ in early-stage AD. Using electrophysiology (patch-clamping) we analyzed the changes in the functional activity of mPFC pyramidal neurons during early-stage AD, in the absence of Aβ plaques, in young 3xTg-AD mice.

## 2 Materials and methods

### 2.1 Animals and tissue preparation

Young male 3xTg-AD and non-Tg B6 (WT) mice (The Jackson Laboratory) at the age of 4–5 months (equivalent to humans 25–30-year-old) were group-housed at the Rush University Medical Center animal facility on a 12 h light/dark cycle. Food and water were available *ad libitum*. Animal care and use procedures were conducted in accordance with NIH, USDA and institutional guidelines, and approved by the Institutional Animal Care.

### 2.2 Whole-cell patch-clamp electrophysiology

The procedure was as descripted elsewhere (Ghatak et al., 2019; Javonillo et al., 2022). Briefly, the mice were deeply sedated with inhalational isoflurane, then perfused transcardially with ice-cold solution (in mM: 248.3 sucrose, 3 KCl, 2 MgSO4, 1.25 NaH2PO4, 26 NaHCO3, 0.1 CaCl2.2H2O, 10 glucose; pH 7.4–7.45, with 335–345 mOsm) containing 3 mM kynurenic acid and 1 mM ascorbic acid. Coronal brain sections (280 μm) containing the mPFC were sliced and transferred to a holding chamber containing oxygenated (95% O2/5% CO2) artificial cerebrospinal fluid (aCSF; in mM: 125 NaCl, 2.5 KCl, 25 NaHCO3, 1.25 NaH2PO4, 1 MgCl_2_.6H2O, 2 CaCl2.2H2O, and 15 glucose; pH 7.4–7.45, with 305–315 mOsm) containing 1 mM ascorbic acid.

After ∼1 h incubation, slices were moved to a recording chamber perfused with oxygenated aCSF. Glass electrodes were pulled and filled with an internal solution which had a resistance of 4–6 MΩ and were used for patch-clamp recording. Different pipette solutions were used optimized for the activity of interest. For evoking action potentials (in mM): 120 K-gluconate, 10 HEPES, 20 KCl, 2 MgCl_2_.6H2O, 3 Na2ATP, 0.3 NaGTP, and 0.1 EGTA; pH: 7.3–7.35; osmolarity: 280–285 mOsm; For sEPSC and sIPSC recording (in mM): 135 CsOH, 5 CsCl, 10 HEPES, 2 MgCl_2_.6H2O, 3 Na2ATP, 0.3 NaGTP; pH: 7.30; osmolarity: 280–285 mOsm. For recording of VGCC activity (in mM): 140 CsOH, 10 HEPES, 2 MgCl_2_.6H2O, 3 Na2ATP, 0.3 NaGTP; pH: 7.30; osmolarity: 280–285 mOsm. The slices were constantly perfused with oxygenated aCSF of the same composition as in the holding chamber, with the exception while assessing spontaneous excitatory and inhibitory postsynaptic currents (sEPSCs and sIPSCs, respectively), in which Mg^2+^ was excluded to remove external blockade of NMDA receptors by Mg^2+^.

Glutamatergic pyramidal neurons in layer V-VI of the mPFC were visually identified using Nikon ECLIPSE E600FN microscope. The acquisition system consisted of Axon Digidata 1,550 analog-digital converter and Multiclamp 700B amplifier. Signals were low-pass filtered at 10 kHz except for the sEPSC/sIPSCs which were filtered at 3 kHz. All neurons from non-Tg B6 (WT) and 3xTg-AD mice met the criteria of the RMP that was equal or more hyperpolarized than −60 mV, and the amplitude of action potential (AP, firing) was ≥60 mV. The protocol for evoking action potentials included a series of 500 ms current pulses with the intensity ranging from −400 to +400 pA with a 25-pA increment. The sEPSCs and sIPSCs were assessed at −70 mV and 0 mV gap-free respectively in the voltage-clamp mode. We also blocked ionotropic glutamatergic AMPA and NMDA receptors using 10 µM NBQX and 50 µM D-AP5, respectively. The membrane potential (*V*m) rebound following the end of *V*m hyperpolarization was assessed to determine functional activity of low voltage-gated Ca^2+^ channels in neurons (Napier et al., 2014; Khodr et al., 2016; Wayman et al., 2016). Ca^2+^ influx via high voltage-activated (HVA) Ca^2+^ channels (VGCCs) were evoked with a 40 ms rheobase (the minimal depolarizing current pulse that evoked voltage-sensitive Ca^2+^ spike) in the presence of 0.5 µM tetrodotoxin (TTX), 20 mM tetraethyl ammonium (TEA), 2.5 mM kynurenic acid, and 100 µM picrotoxin to block intrinsic/synaptic activity that could affect Ca^2+^ influx.

The effects of Aβ42 on firing of living mPFC pyramidal neurons were assessed in the brain slices from young 3xTg-AD mice and age-matched WT mice. Aβ42 (25 and 50 nM) was applied in the bath. A previous study demonstrated that the levels of Aβ42 in the cerebrospinal fluid (CSF) of AD patients at the earliest stage are in the range of 136–234 ng/L (up to proximately 50 pM) (Palmqvist et al., 2017). Although the exact levels of Aβ42 in the cortical regions of AD patients at late stage are unknown, we used higher concentrations of Aβ42 [e.g., 25 and 50 nM; that were ∼500–1,000-fold higher than that found in CSF of AD patients at their earliest stage (Palmqvist et al., 2017)], to determine if and how Aβ42 might affect functional activity of alive cortical neurons in the brain.

### 2.3 Data analysis and statistics

We used Clampex 11 to acquire all electro-physiological data. The features of action potentials and Ca^2+^ spikes were analyzed using Clampfit 11. The sEPSC/sIPSC properties were assessed using the template method in Axograph, where post-event detection criteria (amplitude, rise time and decay time constant) were used to filter out non-characteristic events. Prism 7 (GraphPad Software Inc., La Jolla, CA) was used for statistical analysis. Two-way repeated measures (rm) ANOVA were used to analyze the spike number vs. current relationship followed by Sidak’s *post hoc* test, to determine the effects of genotype on firing. Membrane properties, sEPSC/sIPSC properties, and Ca^2+^ spike features were analyzed using Student’s t-test. One-way ANOVA was used to compare the effects of specific glutamatergic AMPA and NMDA receptor antagonists in each genotype followed by Tukey’s *post hoc* test. Two sample Kolmogorov-Smirnov (K-S) tests were used to compare the cumulative fraction distribution of the amplitudes and inter-event intervals (IEI) of sEPSCs and sIPSCs in neurons. Statistical significance was set at *p* < 0.05. The outlier(s) was excluded if the value of which was greater than 2-fold of the standard deviation from the mean. All data were expressed as mean ± standard error (SE).

## 3 Results

### 3.1 Glutamatergic mPFC pyramidal neurons are hyperactivated in early stage of AD

**FIGURE 1:**
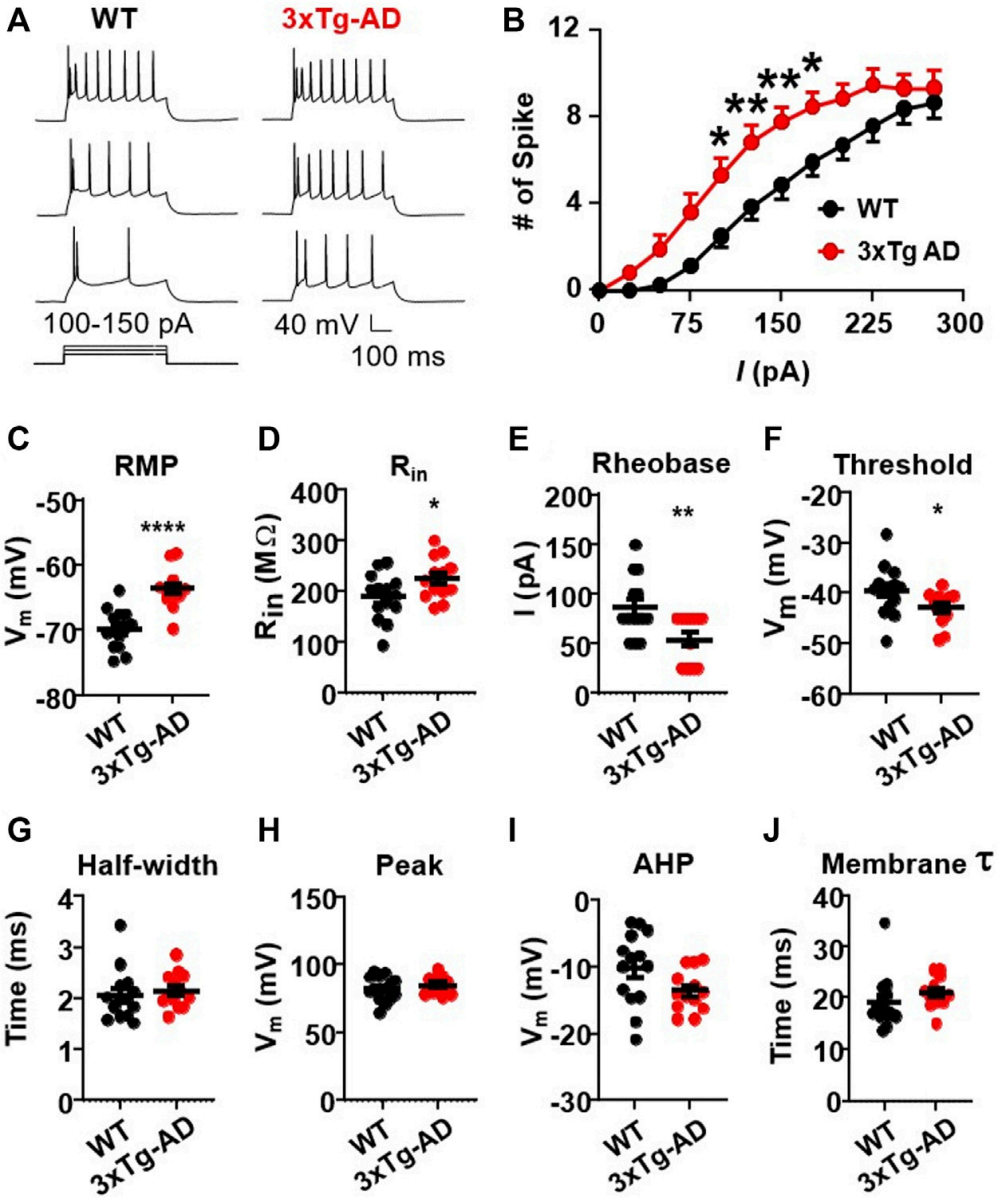
The firing activity of mPFC pyramidal neurons is significantly increased in young 3xTg-AD mice compared to age-matched WT mice. **(A)** Sample spike traces from young WT and 3xTg-AD mice at age of ∼4 months. **(B)** The firing (spike) number was significantly increased in mPFC neurons of young 3xTg-AD mice compared to age-matched WT mice (*, **p < 0.05, 0.01). **(C)** The resting membrane potential (RMP) was significantly depolarized in mPFC neurons of 3xTg-AD mice compared to WT mice: ****p < 0.0001). **(D)** The input resistance (R_in_) was significantly higher in mPFC neurons from 3xTg-AD mice compared to those from WT mice: *p = 0.0398). (**E,F)** The rheobase and firing threshold were significantly lower in mPFC neurons of 3xTg-AD mice compared to WT controls (**p = 0.0065 and *p = 0.0496, respectively). **(G–J)** There were no significant changes in the AP half-width, AP peak, AHP, and membrane time constant *tau* in mPFC neurons of 3xTg-AD mice (black: *n* = 14 neurons from 9 WT mice; red: *n* = 13 neurons from 9 3xTg-AD mice).

We first assessed evoked action potentials of mPFC pyramidal neurons in WT or 3xTg-AD mice at young age (≤5 months, equivalent of ≤30-year-old humans) using positive current step pulses (500 ms, 25–300 pA) that mimicked excitatory inputs. We found that firing (the number of evoked action potentials) of mPFC neurons was significantly increased in 3xTg-AD mice compared to those from WT mice (3xTg-AD vs. WT: *n* = 13 neurons/9 mice vs. 14 neurons/9 mice; Two-way rm ANOVA: genotype effect: *F(1,25)* = 7.713, *p* = 0.0102; current effect: *F(11,275)* = 135.8, *p* < 0.0001; interaction: *F(11,275)* = 2.943, *p* = 0.0011; Sidak’s *post hoc* test: *,**p < 0.05, 0.01. Figures 1A, B).

This neuronal hyperactivity identified in 3xTg-AD mice was corroborated with alterations in their membrane properties, including a significant depolarization of the resting membrane potentials (RMP, 3xTg-AD vs. WT: *n* = 13 neurons/9 mice vs. 14 neurons/9 mice, −63.71 ± 0.83 mV vs. −69.90 ± 0.81 mV; unpaired *t*-test: *t25* = 5.350, ****p < 0.0001. Figure 1C), and an increase of the input resistance (Rin, 3xTg-AD vs. WT: *n* = 13 neurons/9 mice vs. 14 neurons/9 mice, 225.6 ± 11.44 MΩ vs. 189.2 ± 12.22 MΩ; unpaired *t*-test: *t25* = 2.169, *p* = 0.0398. Figure 1D). Additionally, the rheobase required for evoking the initial action potential was also significantly reduced in mPFC neurons from 3xTg-AD mice compared to those in WT mice (3xTg-AD vs. WT: *n* = 13 neurons/9 mice vs. 14 neurons/9 mice, 53.85 ± 6.85 pA vs. 85.71 ± 8.17 pA; unpaired *t*-test: *t25* = 2.966, **p = 0.0065. Figure 1E), while their firing threshold was significantly decreased (3xTg-AD vs. WT: *n* = 13 neurons/9 mice vs. 14 neurons/9 mice, −42.85 ± 0.90 vs. −39.49 ± 1.33 mV; unpaired *t*-test: *t25* = 2.064, *p = 0.0496. Figure 1F).

There was no change in the half-width of action potential (3xTg-AD vs. WT: *n* = 13 neurons/9 mice vs. 14 neurons/9 mice, 2.15 ± 0.09 ms vs. 2.05 ± 0.13 ms, *p* > 0.05, Figure 1G), time constant (3xTg-AD vs. WT: *n* = 13 neurons/9 mice vs. 14 neurons/9 mice, 20.97 ± 0.81 ms vs. 19.14 ± 1.38 ms; unpaired *t*-test: *t25* = 1.122, *p* = 0.2725. Figure 1H), peak (3xTg-AD vs. WT: *n* = 13 neurons/9 mice vs. 14 neurons/9 mice, 84.64 ± 1.75 mV vs. 82.12 ± 2.49 mV; unpaired *t*-test: *t25* = 0.8164, *p* = 0.422. Figure 1I), and afterhyperpolarization potentials (AHP, 3xTg-AD vs. WT: *n* = 13 neurons/9 mice vs. 14 neurons/9 mice, −13.59 ± 0.86 mV vs. −10.16 ± 1.46 mV; unpaired *t*-test: *t25* = 1.984, *p* = 0.0584. Figure 1J) in neurons from young 3xTg-AD mice compared to those in age-matched WT mice.

These results reveal that in the context of early-stage AD, young mPFC pyramidal neurons are abnormally *hyperactive* in response to excitatory stimuli. Whether aging worsens such neuronal dysfunction requires further investigation.

### 3.2 Spontaneous excitatory synaptic neurotransmission is significantly enhanced in mPFC pyramidal neurons in early stage of AD

Glutamatergic mPFC pyramidal neurons receive excitatory inputs from the ipsilateral and contralateral mPFC regions, as well as hippocampus and some other regions that are critical for neurocognition (Buckner et al., 2005; Palmqvist et al., 2017). These excitatory synaptic inputs are mainly glutamatergic, which initially activate ionotropic AMPARs and NMDARs, and consequentially induce membrane depolarization of postsynaptic mPFC neurons, which activates voltage-gated sodium (Na^+^), K^+^ and Ca^2+^ channels; and ultimately generates action potentials (firing). To determine if excitatory synaptic inputs to the mPFC alter neuronal activity in the early stages AD, we evaluated the spontaneous excitatory postsynaptic currents (sEPSCs) in such hyperactive mPFC neurons.

**FIGURE 2:**
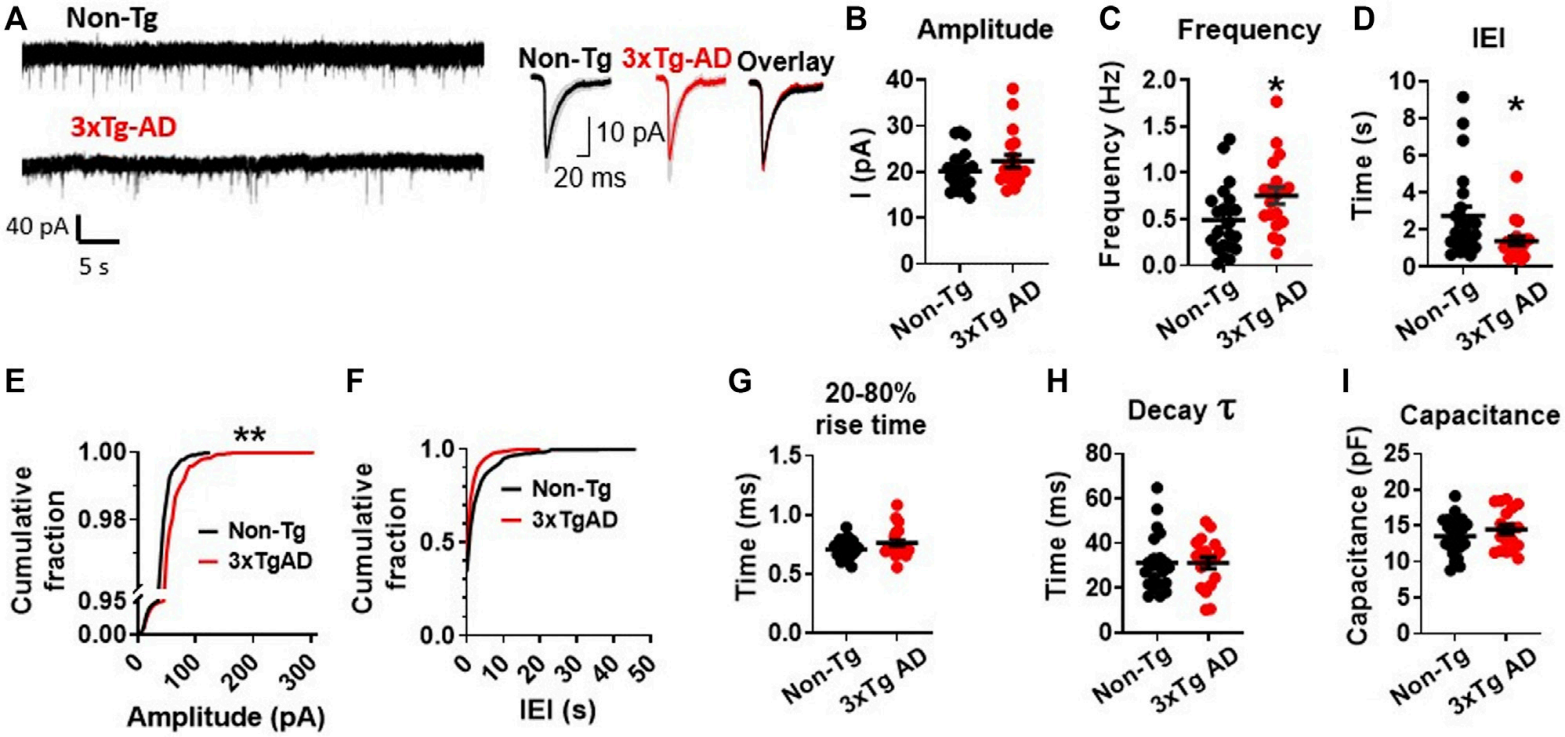
Early-stage AD induces a significant increase in spontaneous excitatory synaptic activity among mPFC pyramidal neurons in young 3xTg-AD mice compared to age-matched WT controls. **(A)** Representative sEPSC recording from non-Tg and 3xTg-AD neurons (left panel). The averaged sEPSC events (right panel) from young WT (left) and 3xTg-AD (middle) mice, and the overlaid averaged traces of sEPSCs from both groups (right). The shading represents the SD. **(B)** There was a trend of increase in the amplitude (peak, pA) of sEPSCs in mPFC neurons from 3xTg-AD mice. Each circle depicts the average amplitude of sEPSCs from each sampled cell. **(C)** The frequency (Hz) of sEPSCs was significantly higher in mPFC neurons of 3xTg-AD mice compared to WT mice (*p = 0.0342). **(D)** Inter-event interval (IEI) was significantly shorter in neurons of 3xTg-AD mice compared to WT mice (*p = 0.0244). Each circle depicts the average IEI from each sampled cell. **(E)** The cumulative distribution fraction of sEPSC amplitudes was significantly different in mPFC neurons from 3xTg-AD compared to WT controls (**p = 0.0019). All individual sEPSC events from each sampled cell (as shown in Supplementary Figure S1A) were used to plot the amplitude cumulative fraction distribution. **(F)** There was no significant change in the cumulative distribution fraction of IEI between WT and 3xTg-AD mice. All individual sEPSC events from each sampled cell (as shown in Supplementary Figure S1B) were used to plot the IEI cumulative fraction distribution. There was also no significant difference in the 20-80% rise time **(G)**, decay time constant **(H)**, and cell capacitance **(I)** in neurons between WT and 3xTg-AD mice (black: *n* = 22 neurons from 8 WT mice; red: *n* = 19 neurons from 7 3xTg-AD mice).

During a 300 s long gap-free recording with a holding potential *V*_h_ = −70 mV, we found no significant change in the amplitudes of sEPSCs in mPFC neurons from young 3xTg-AD mice compared to those from WT mice (3xTg-AD vs. WT: *n* = 19 neurons/7 mice vs. 22 neurons/8 mice, 21.47 ± 1.13 pA vs. 20.09 ± 0.90 pA; unpaired *t*-test: *t*_*38*_ = 0.9689, *p =* 0.3387. Figures 2A, B; Supplementary Figure S1A). However, the frequency of sEPSC events was significantly increased (3xTg-AD vs. WT: *n* = 19 neurons/7 mice vs. 22 neurons/8 mice, 0.75 ± 0.09 Hz vs. 0.49 ± 0.08 Hz; unpaired *t*-test: *t*_*38*_ = 2.096, *p = 0.0428. Figure 2C), and the inter-event intervals (IEI) among sEPSCs was significantly decreased (3xTg-AD vs. WT: *n* = 19 neurons/7 mice vs. 22 neurons/8 mice, 1.41 ± 0.25 s vs. 2.76 ± 0.50 s; unpaired *t*-test: *t*_*38*_ = 2.244, *p = 0.0308. Figure 2D; Supplementary Figure S1B) in mPFC neurons from 3xTg-AD compared to WT mice. Moreover, we also found that the probability of larger amplitude sEPSC events (100–300 pA) was significantly greater in neurons from 3xTg-AD mice compared to WT mice (Two sample K-S test: *D* = 0.4433, **p = 0.0019. Figure 2E; Supplementary Figure S1A).

Meanwhile, there was also no significant change in the fraction distribution of IEI (Two sample K-S test: *D* = 0.2644, *p* = 0.2621. Figure 2F; Supplementary Figure S1B), the 20%–80% rise time (3xTg-AD vs. WT: *n* = 19 neurons/7 mice vs. 22 neurons/8 mice, 0.77 ± 0.03 ms vs. 0.71 ± 0.02 ms; unpaired *t*-test: *t*_*38*_ = 1.726, *p* = 0.0925. Figure 2G), the decay time constant (3xTg-AD vs. WT: *n* = 19 neurons/7 mice vs. 22 neurons/8 mice, 31.56 ± 2.695 ms vs. 31.23 ± 2.695 ms; unpaired *t*-test: *t*_*38*_ = 0.08624, *p* = 0.9317. Figure 2H), and the cell capacitance (indicative of cell size) (3xTg-AD vs. WT: *n* = 19 neurons/7 mice vs. 22 neurons/8 mice, 14.44 ± 0.68 pF vs. 13.54 ± 0.57 pF; unpaired *t*-test: *t*_*38*_ = 1.021, *p* = 0.3135. Figure 2I), among mPFC neurons in 3xTg-AD mice compared to those from WT mice.

The significantly-increased probability for larger sEPSC (greater amplitudes) events (Figure 2E), in combination with the significantly-increased frequency of sEPSCs (Figure 2B), indicates substantial enhancement in the excitatory pre-/post-synaptic activity of young mPFC pyramidal neurons in early-stage AD.

### 3.3 Blockade of AMPA receptors and NMDA receptors eliminates increased frequency of sEPSCs in mPFC pyramidal neurons in early stage of AD

**FIGURE 3:**
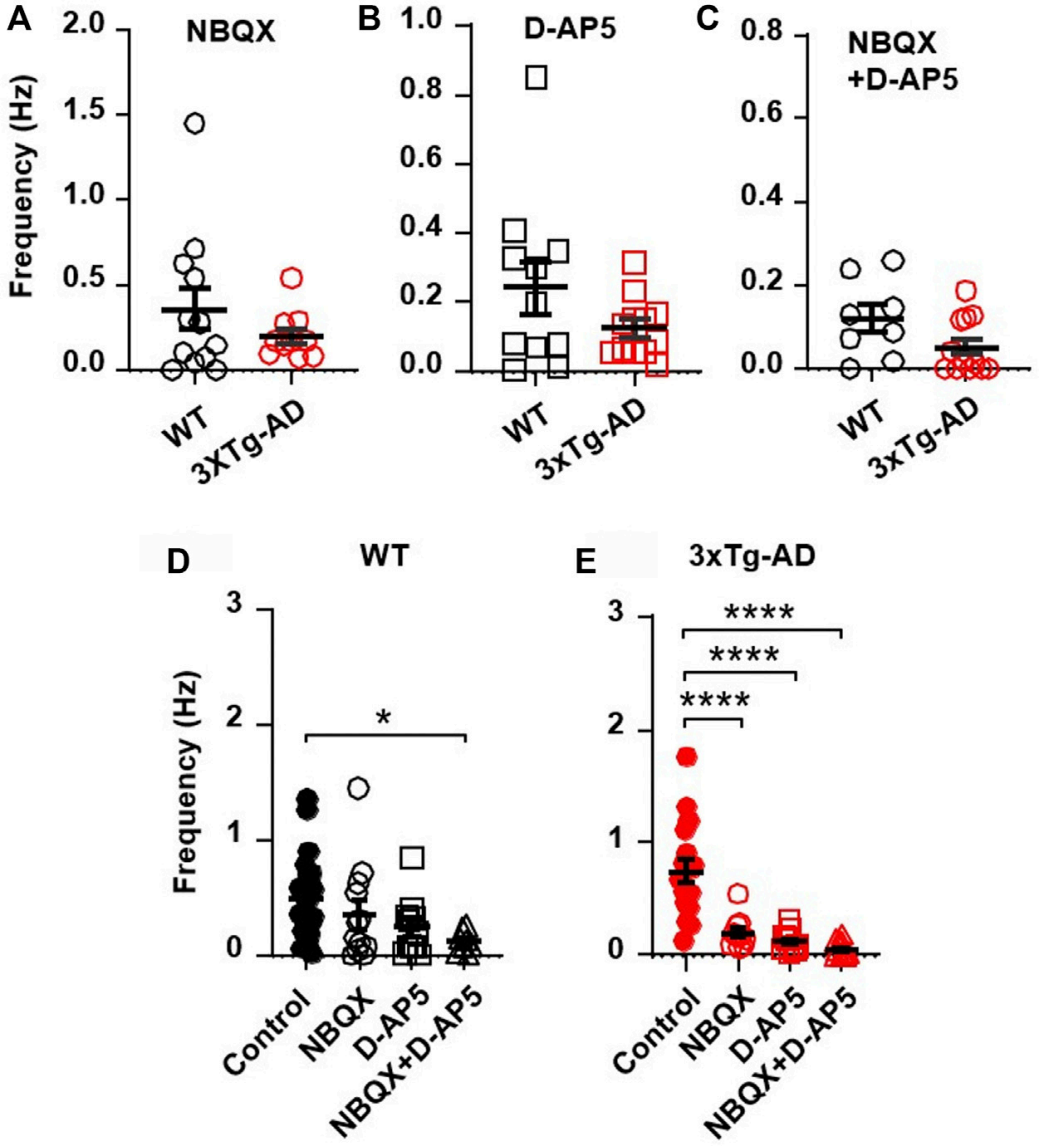
Blockade of AMPARs and NMDARs eliminates the abnormally increased sEPSC frequency in mPFC neurons of young 3xTg-AD mice. (**A,B)** Blockade of AMPARs (with NBQX, 10µM; **(A)** or NMDARs (with D-AP5, 50µM; **(B)** significantly reduced sEPSCs in neurons of young mice, regardless of genotype. **(C)** There was no significant difference in sEPSCs of mPFC neurons between WT and 3xTg-AD mice following combined blockade of AMPARs/NMDARs. **(D)** Co-blockade of AMPARs and NMDARs (open triangles; *n* = 8 neurons/4 mice) completely abolished sEPSCs in mPFC neurons from WT mice (basal control vs. NBQX + D-AP5, *p = 0.0421). **(E)** Co-blockade of AMPARs/NMDARs also eliminated sEPSCs in mPFC neurons of 3xTg-AD mice (basal control vs. NBQX/D-AP5/NBQX + D-AP5 treatment; all ****p < 0.0001).

To determine if the increased excitatory synaptic activity was mediated by presynaptic release of glutamate and postsynaptic glutamatergic receptors, we blocked AMPARs and/or NMDARs and evaluated the sEPSCs thereafter. We found that blocking AMPARs (by NBQX, a selective AMPAR antagonist) and NMDARs (D-AP5, a selective NMDAR antagonist) significantly reduced the previously-increased sEPSC frequency in neurons from 3xTG-AD mice compared to WT mice (NBQX, 10µM, 3xTg-AD vs. WT: *n* = 11 neurons/7 mice vs. 12 neurons/8 mice, 0.20 ± 0.04 Hz vs. 0.36 ± 0.12 Hz; unpaired *t*-test: *t*_*21*_ = 1.177, *p* = 0.2522. Figure 3A; and D-AP5, 50µM, *n* = 12/6 mice vs. 11/3 mice**,** 0.12 ± 0.03 Hz vs. 0.24 ± 0.07 Hz; unpaired *t*-test: *t*_*21*_ = 1.566, *p* = 0.1323; Figure 3B). Combined blockade of AMPARs/NMDARs abolished the previously-increased sEPSC frequency (3xTg-AD vs. WT: NBQX 10 µM + D-AP5 50 µM: *n* = 12 neurons/7 mice vs. 8 neurons/4 mice, 0.05 ± 0.02 vs. 0.12 ± 0.03; unpaired *t*-test: *t*_*18*_ = 1.868, *p* = 0.0781. Figure 3C). Comparison across the different treatments revealed that, regardless of the genotype, combined blockade of AMPAR/NMDAR abolished sEPSCs in these neurons (One-way ANOVA: WT: *F*_*(3,49)*=_2.17, *p* = 0.0421; Tukey’s *post hoc* test: *p = 0.0464, Figure 3D; 3xTg-AD: *F*_*(3,49)*_ = 8.89, ****p < 0.0001; Tukey’s *post hoc* test: ****p < 0.0001, Figure 3E).

Together, these results indicate that an abnormal increase in the presynaptic glutamate release and the postsynaptic AMPAR/NMDAR activity plays a crucial role in inducing abnormally enhanced excitatory synaptic neurotransmission and hyperactivity of mPFC pyramidal neurons in the early stage of AD.

### 3.4 Spontaneous inhibitory synaptic activity of mPFC pyramidal neurons is unchanged in early AD stages

Neuronal excitability in a brain network is defined by its excitation/inhibition balance. Thus, we also analyzed the inhibitory presynaptic inputs to mPFC neurons and the responses of them during the early stage of AD. Using a holding potential of *V*_h_ = 0 mV, we assessed the spontaneous inhibitory postsynaptic currents (sIPSCs) in postsynaptic mPFC neurons during a 300s long gap-free protocol.

**FIGURE 4:**
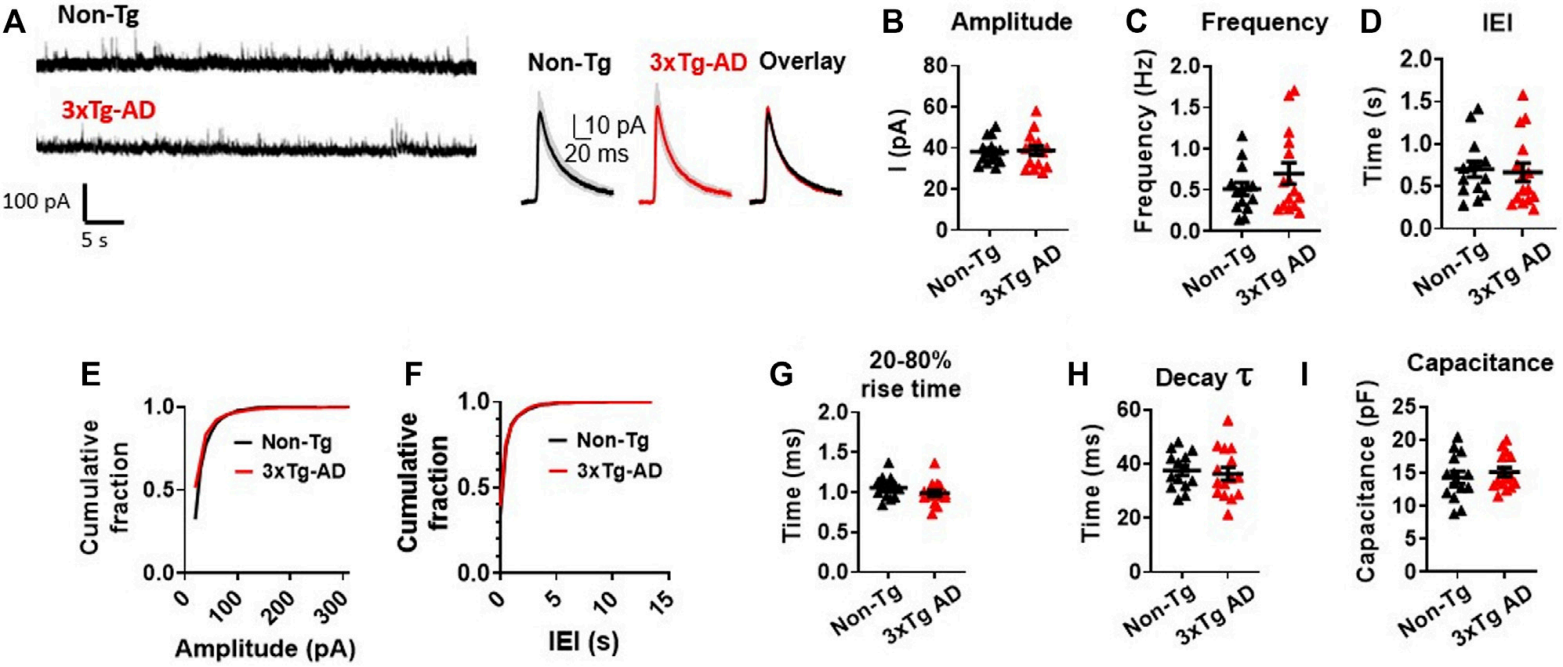
Spontaneous inhibitory synaptic activity of mPFC pyramidal neurons is unchanged in young 3xTg-AD mice compared to WT mice. **(A)** Representative sIPSC recording from non-Tg and 3xTg-AD neurons (left panel). The averaged sIPSC events (right panel) from mPFC neurons of 3–5 months WT (left) and 3xTg-AD (middle) mice and overlaid averaged traces from both groups (right). The shading represents the SD. **(B-I)** Each circle depicts the average respective property of sIPSCs from each sampled cell **(B–D, G–I)**. In the amplitude and IEI cumulative fraction distribution, all individual sIPSC events from each sampled cell (as in Supplementary Figure S2) have been evaluated **(E,F)**. There was no significant difference in the amplitude; frequency; IEI; cumulative fraction distribution of sIPSC amplitudes; cumulative fraction distribution of IEI; 20%–80% rise time (ms); decay time constant of the sIPSCs; and cell capacitance (pF) of sIPSCs in mPFC neurons between young WT and 3xTg-AD mice (all *p* > 0.05, black: *n* = 14 neurons/6 WT mice vs. red: *n* = 15 neurons/8 3xTg-AD mice).

The amplitude of the sIPSCs were found to be very similar in mPFC neurons from 3xTg-AD mice as compared to those of WT mice (3xTg-AD vs. WT: *n* = 15 neurons/8 mice vs. 14 neurons/6 mice, 38.70 ± 2.20 pA vs. 38.22 ± 1.67 pA; unpaired *t*-test: *t*_*27*_ = 0.1712, *p =* 0.8654. Figures 4A, B; Supplementary Figure S2A). Unlike the sEPSCs, there was no significant difference in the frequency (3xTg-AD vs. WT: *n* = 15 neurons/8 mice vs. 14 neurons/6 mice, 0.70 ± 0.13 Hz vs. 0.51 ± 0.08 Hz; unpaired *t*-test: *t*_*27*_ = 1.230, *p* = 0.2293. Figure 4C), and IEI (3xTg-AD vs. WT: *n* = 15 neurons/8 mice vs. 14 neurons/6 mice, 0.67 ± 0.11 s vs. 0.70 ± 0.09 s; unpaired *t*-test: *t*_*27*_ = 0.2626, *p* = 0.7949. Figure 4D; Supplementary Figure S2B) of sIPSCs among mPFC neurons between 3xTg-AD and WT mice.

There was also no significant difference in the cumulative fraction distribution of the event amplitudes and the IEI in mPFC neurons between the two genotypes (Two sample K-S test: amplitude: *D* = 0.1858, *p* = 0.6963. Figure 4E; Supplementary Figure S2A; IEI: *D* = 0.2357, *p* = 0.5358; Figure 4F; Supplementary Figure S2B), as well as in the sIPSCs’ 20%–80% rise time (in ms: 3xTg-AD vs. WT: *n* = 15 neurons/8 mice vs. 14 neurons/6 mice, 0.99 ± 0.04 ms vs. 1.06 ± 0.04 ms; unpaired *t*-test: *t*_*27*_ = 1.326, *p* = 0.1959. Figure 4G), the decay time constant (in ms: 3xTg-AD vs. WT: *n* = 15 neurons/8 mice vs. 14 neurons/6 mice, 36.26 ± 2.40 vs. 37.47 ± 1.80 ms; unpaired *t*-test: *t*_*27*_ = 0.4000, *p* = 0.6923. Figure 4H), and the cell capacitance (3xTg-AD vs. WT: *n* = 15 neurons/8 mice vs. 14 neurons/6 mice, 15.10 ± 0.69 ms vs. 14.27 ± 0.94 ms; unpaired *t*-test: *t*_*27*_ = 0.7197, *p* = 0.4779. Figure 4I), between the two mouse genotypes accordingly.

These results suggest that unlike abnormal increase in the spontaneous excitatory postsynaptic activity, the spontaneous inhibitory postsynaptic activity of mPFC neurons was not significantly altered during early stage of AD.

### 3.5 Ca^2+^ influx through voltage-gated Ca^2+^ channels (VGCCs) are unchanged in mPFC pyramidal neurons at early AD stages

Neuron’s excitability is regulated by interactive synaptic/intrinsic activity, in which intracellular Ca^2+^ homeostasis ([Ca^2+^]_in_) plays a critical role. Besides NMDARs, VGCCs (including *high* and *low* voltage-activated Ca^2+^ channels) also contribute to maintaining [Ca^2+^]_in_. VGCC dysfunction (including Aβ-formed Ca^2+^ channels) underlies several neurodegenerative diseases, including but not limited to AD (Arispe et al., 1993; Pollard et al., 1993; Abramov et al., 2004; Lal et al., 2007; Shirwany et al., 2007; Itkin et al., 2011), neuroAIDS (Hu, 2016; Hu, 2020), and substance use disorders (Ford et al., 2009; Wayman et al., 2012; Wayman et al., 2016). Thus, we explored VGCC activity in mPFC neurons at early-stage AD modeled in young 3xTg-AD mice.

**FIGURE 5:**
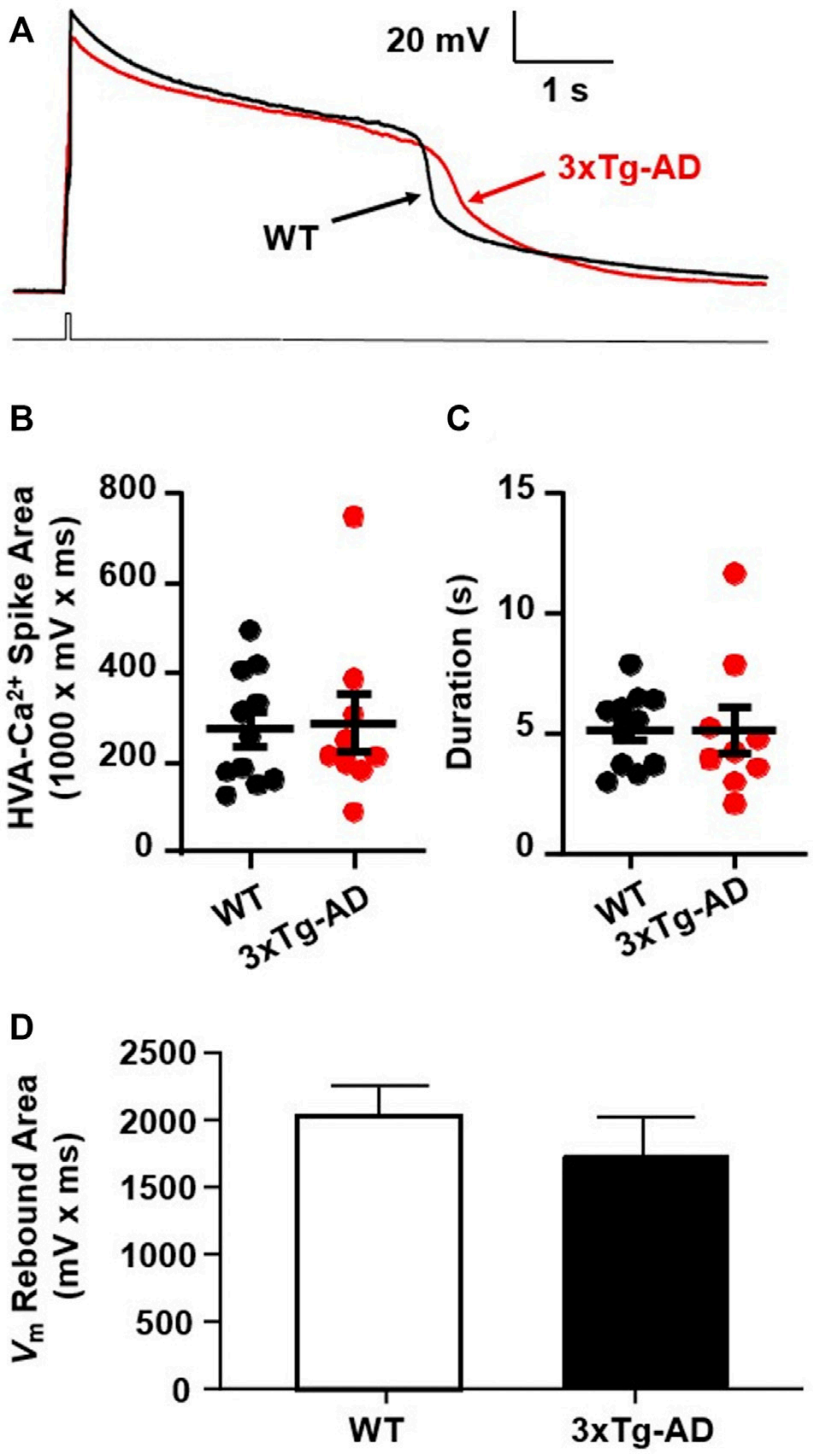
There was no meaningful increase in Ca^2+^ influx via VGCCs into mPFC neurons in young 3xTg-AD mice compared to WT mice. **(A)** Ca^2+^ spike in mPFC neurons from ∼4 months WT (black) and 3xTg-AD (red) mice in response to a 40 ms rheobase step current pulse. **(B,C)** There was no significant difference in the total area (mV x ms) or duration of Ca^2+^ spikes (s) in neurons between WT and 3xTg-AD mice (both *p* > 0.05, black: *n* = 11 neurons/7 WT mice vs. red: *n* = 9 neurons/6 3xTg-AD mice). **(D)** The *V*_m_ rebound area was not significantly changed in mPFC neurons from 4 months 3xTg-AD mice as compared to those from WT controls (*n* = 14 vs. 13 neurons, *p* > 0.05).

High voltage-activated Ca^2+^ spikes were evoked in mPFC neurons with a 40 ms rheobase current step pulse that depolarized *V*m. To eliminate the influence of voltage-sensitive Ca^2+^ influx by non-VGCC sources, firing and synaptic activity mediated by other voltage-sensitive membrane ion channels and glutamatergic receptors were all blocked concurrently using selective blockers/antagonists for Na + channels (tetrodotoxin, TTX, 0.5 µM), K+ channel blockers (tetraethyl ammonium, TEA, 20 mM), glutamate receptors (kynurenic acid, 2.5 mM), and GABAA receptors (picrotoxin, 100 µM), respectively. The total area and duration of the resulting Ca^2+^ spikes (represented in Figure 5A) were used to indicate the levels of calcium influx.

We found that neither the area nor duration of evoked Ca^2+^ spikes was significantly changed in mPFC neurons from young 3xTg-AD mice as compared to WT mice (3xTg-AD vs. WT, *n* = 9 neurons/6 mice vs. 11 neurons/7 mice; the total area: 288.3 ± 3.89 mV × ms vs. 275.6 ± 8.01 mV × ms, unpaired *t*-test: *t*_*18*_ = 0.1787, *p* = 0.8602. Figure 5B; and the duration: 5.13 ± 0.98 s vs. 5.16 ± 0.48 s, unpaired *t*-test: *t*_*18*_ = 0.0265, *p* = 0.9791; Figure 5C). In addition, activity of low voltage-activated Ca^2+^ channels, indicated by the *V*_m_ rebound immediately following the end of membrane hyper-polarization, was not significantly changed in mPFC neurons from young 3xTg-AD mice compared to WT controls (*n* = 13 vs. 14 neurons, *p* > 0.05. Figure 5D).

These results suggest that at *early-stage* AD, Ca^2+^ influx via VGCCs has not been significantly altered yet in *young* mPFC neurons. However, given that Aβ plaques and Ca^2+^-permeable channels are not formed at early-stage AD, significant dysfunction of VGCCs is expected to occur at later stages of AD during aging.

### 3.6 Acute Aβ42 exposure significantly increases firing of mPFC neurons in early-stage AD, but not in age-matched non-Tg controls

The formation of Aβ42 plaque is thought to be a hallmark of AD beginning with Aβ42 deposition in the extracellular matrix around neurons in the AD brain in its late stage. As depicted in Figure 1, mPFC neurons are hyperactive even at early-stage AD. However, it is unknown if, how, and to what extent Aβ42 alters activity of cortical neurons in the brain. To determine if acute Aβ42 affects mPFC neurons’ firing and further enhance mPFC neuronal hyperactivity during early-stage AD, we evaluated firing of mPFC neurons in response to acute Aβ42 exposure *ex vivo* (in bath) for ∼10 min.

**FIGURE 6:**
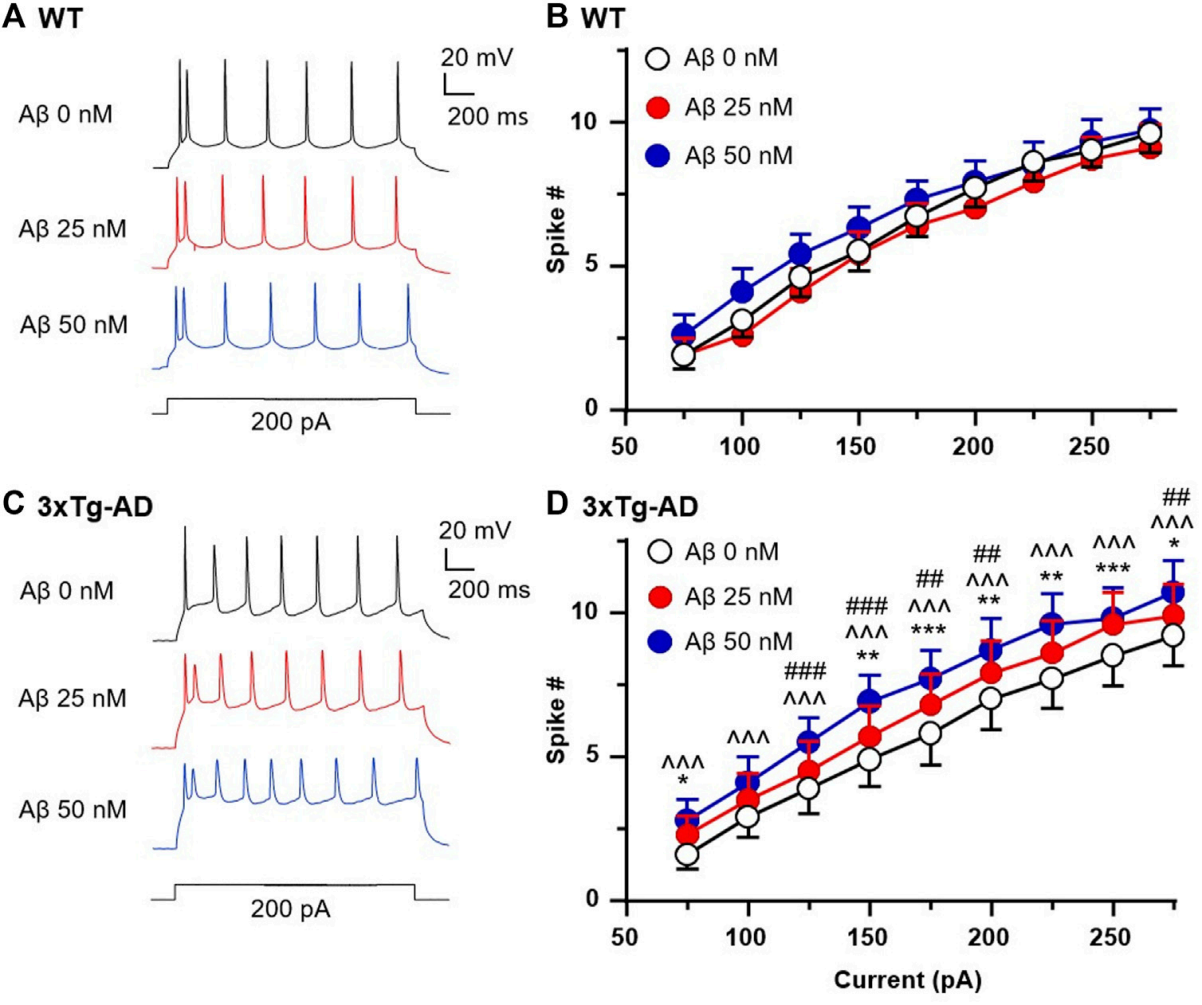
Acute Aβ42 treatment *ex vivo* elevates firing of hyperactive mPFC pyramidal neurons in young 3xTg-AD mice, but not in neurons from age-matched WT mice. Sample spike traces from young WT **(A)** and 3xTg-AD mice **(C)** at age of 4–5 months with increasing acute dose of Aβ42. **(B)** Bath application of Aβ42 peptide (25 or 50 nM, both *p* > 0.05) did not affect firing of mPFC neurons in young WT mice (*n* = 10 neurons/8 mice). **(D)** But the firing of mPFC neurons was significantly increased in age-matched 3xTg-AD mice (*n* = 10 neurons/7 mice) following such acute Aβ42 treatment in a dose-dependent manner (dose effect: *F(2,18)* = 5.766, *p* = 0.0116; *post hoc* test: 25 nM vs. control: *,**,***p < 0.05, 0.01, 0.001; 50 nM vs. control: ^^^*p* < 0.001; 25 nM vs. 50nM: ^##^,^###^ *p* < 0.01,0.001).

We found that the number of spikes evoked in mPFC neurons in young WT mice was not altered by 25 and 50 nM Aβ42 compared to basal control with vehicle perfusion (aCSF, 0nM, *n* = 10 neurons/8 mice, Two-way rm ANOVA: dose effect: *F(2,18)* = 2.221, *p* = 0.1374; current effect: *F(8,72)* = 333.1, *p* < 0.0001; interaction: *F(16,144)* = 1.626, *p* = 0.0690. Figures 6A, B). In contrast, 25 and 50 nM Aβ42 significantly increased firing in a dose-dependent manner in 3xTg-AD mice (*n* = 10 neurons/7 mice, Two-way rm ANOVA: dose effect: *F(2,18)* = 5.766, *p* = 0.0116; current effect: *F(8,72)* = 140.2, *p* < 0.0001; interaction: *F(16,144)* = 1.027, *p* = 0.4316; Sidak’s *post hoc* test: 25 nM vs. control: *,**,***p < 0.05, 0.01, 0.001; 50 nM vs. control: ^^^*p* < 0.001; 25 nM vs. 50 nM: ^##^,^###^ *p* < 0.01, 0.001. Figures 6C, D).

**FIGURE 7:**
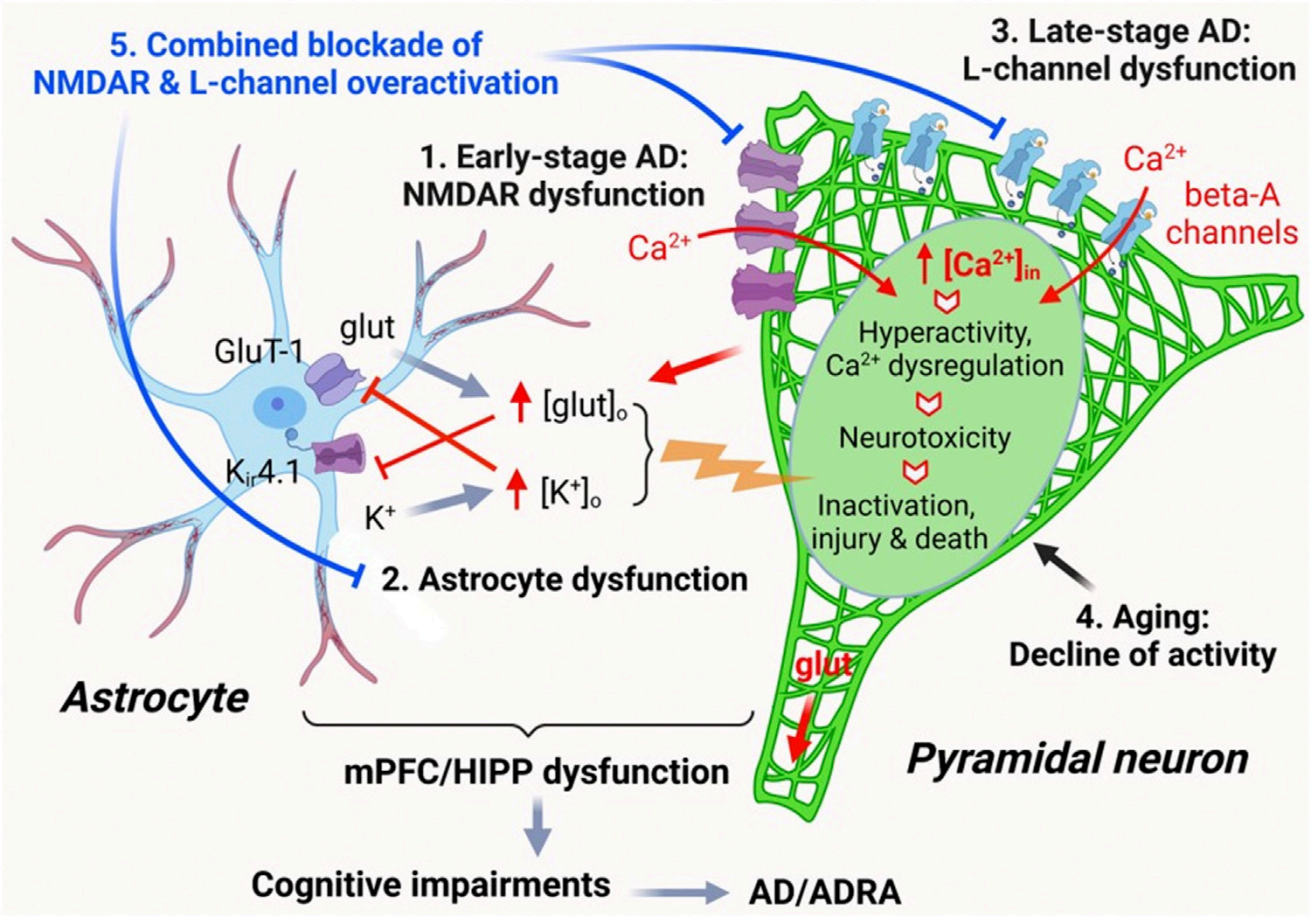
The impact of AD, A_β_, and aging on mPFC pyramidal neuron activity and Ca^2+^ homeostasis. Ca^2+^ homeostasis is essential for the normal function of pyramidal neurons. Neuronal synaptic and intrinsic activity are mediated by various membrane ion channels, including but not limited to NMDARs and L-type Ca^2+^ channels, as well as by astrocytes nearby via regulating glutamate uptake and K^+^ spatial buffering/siphoning. Dysfunction of mPFC pyramidal neurons, including aberrantly-increased firing and excitatory postsynaptic activity mediated by NMDARs/AMPARs, occurs at early-stage AD, without formation of Aβ plaques (1). Such neuronal hyperactivity increases glutamate release and [glut]_o_ in the brain. Increased [glut]_o_ interrupts functional activity of astrocytes in regulating [K^+^]_o_ homeostasis mediated by K_ir_4.1 channels, leading to abnormal increase of [K^+^]_o_, which in turn also inhibits glutamate reuptake by astrocytes. This dysregulation of [glut]_o_/[K^+^]_o_ enhances membrane depolarization of pyramidal neurons, thereby worsening neuronal hyperactivity (2). Aβ is released by mPFC pyramidal neurons and forms plaques in the brain at late-stage AD during aging. Accumulated Aβ also forms Ca^2+^-permeable channels that are similar to L-channels to conducting Ca^2+^ influx, which exacerbates neuronal hyperactivity/Ca^2+^ dyshomeostasis (excessive [Ca^2+^]_in_). Through this final path to neurotoxicity, pyramidal neurons could be driven from overactivation to inactivation, injured, and killed (3). Aging *per se* also induces decline of pyramidal neuron activity and could deteriorate it at later stage AD (4). Combined, but not individual, antagonism of overactive L-channels and NMDARs could significantly alleviate pyramidal neuron function by diminishing hyperactivity and Ca^2+^ dyshomeostasis in the brain of AD (5).

Collectively, these results indicate that Aβ does not affect functional activity of healthy mPFC pyramidal neurons at young age; however, it worsens hyperactivity of these neurons in the context of AD at early-stage. Whether Aβ exacerbates such cortical neuron dysfunction in the brain during the late stage of AD and aging requires further investigation. Here, we propose a working hypothesis that elucidates the mechanism of neuron dysfunction and its interplay with astrocyte dysfunction in the brain of AD in Figure 7.

## 4 Discussion

The present study demonstrates that at the early stage of AD, pyramidal neurons in the mPFC were hyperactive, as indicated by increased firing in young 3xTg-AD mice compared to age-matched controls. This neuronal hyperactivity was mediated by enhanced pre-/post-excitatory synaptic activities, as seen by a higher frequency of sEPSCs, shorter interval between sEPSCs, and increased membrane input resistance. It is worth noting that such neuronal dysfunction occurred in the absence of Aβ deposition, but it was exacerbated when neurons were exposed to Aβ. These findings highlight specific dysfunction of mPFC pyramidal neurons in the early stages of AD.

We also found that blocking AMPARs or NMDARs significantly reduced the previously increased sEPSCs, while antagonizing both receptors completely abolished sEPSCs in mPFC pyramidal neurons, regardless of genotype, and therefore, there was no significant difference in the sEPSCs between 3xTg-AD and non-Tg mice.

The excitatory postsynaptic excitability of neurons is primarily mediated by AMPARs and NMDARs expressed in the neuronal membrane. These ionotropic receptors are activated by the release of glutamate from presynaptic neurons, generating positively charged ion influx; and among them, NMDARs have high permeability to Ca^2+^. Therefore, our results suggest that the heightened sEPSCs in mPFC pyramidal neurons are predominantly induced by an abnormal increase in the presynaptic glutamate release and immediately followed by a consequential enhancement in the postsynaptic AMPAR/NMDAR activity. This augmentation of the excitatory presynaptic and postsynaptic activities drives mPFC pyramidal neurons to fire more frequently and become hyperactive during the early stage of AD (Figure 7). In addition, the excitability of neurons could be enhanced independent of excitatory synaptic factors. Particularly, the depolarized resting membrane potential, increased input resistance, and decreased rheobase and firing threshold of 3xTg-AD neurons suggest changes in intrinsic properties of these neurons. Further exploration, for example, of the functional activity of K_2P_ channels, which play a critical role in maintaining the resting membrane potential, can provide mechanistic insights into these intrinsic changes.

In contrast, the present study also found that the inhibitory postsynaptic activity, indicated by sIPSCs, was not altered in mPFC pyramidal neurons of young 3xTg-AD mice compared to age-matched non-Tg controls. This result suggests that inhibitory neurotransmission, such that mediated by GABA, and the activity of postsynaptic GABA receptors were not significantly impacted in these neurons during the early stage of AD and young age. This uncovering of the imbalance between excitatory and inhibitory synaptic neurotransmission in the mPFC at an early-stage AD requires further investigation to determine if this imbalance persists in the later stage of AD.

Another important finding of the present study is the determination of the effects of Aβ on living mPFC pyramidal neurons in the brain during early-stage AD. Our results indicated that the activity of naïve mPFC pyramidal neurons in young healthy brains was not affected by acute Aβ exposure (see below). The exact Aβ levels in the mPFC, or the effects of Aβ on living cortical neurons in the brain, are challenging to determine because Aβ levels changes dynamically during the progression of AD, decreasing in CSF but increasing in the brain tissues at late-stage AD (Palmqvist et al., 2017). Despite high levels of Aβ_42_ [e.g., 25 and 50 nM, which were ∼500 to 1000-fold higher than those found in the CSF of early Aβ accumulators (Palmqvist et al., 2017)], young cortical neurons did not show any alterations in their firing. However, 25 and 50 nM Aβ_42_, which might mimic the effects of accumulated Aβ, potentiated their hyperactivity in early-stage AD. These results demonstrate that without Aβ plaques and Aβ-mediated L-channel-like Ca^2+^ channels in pyramidal neurons (which are found in the late stage of AD) (Lal et al., 2007; Shirwany et al., 2007; Itkin et al., 2011), Aβ has limited effects on altering the functional activity of live cortical neurons in cognition-regulating brain regions (Figure 7).

3xTg-AD mice carry mutations in the Amyloid Precursor Protein (APP), Microtubule-Associated Protein Tau (MAPT) and presenilin (PSEN-1) genes. In the 3xTg-AD mice, the APP and PSEN-1 mutations assist extracellular Aβ deposits in the cortex by 6 months while tau tangles, owing to MAPT mutation, become evident only after 12 months of age. With the aim to detect cortical changes associated with *early* AD, our study has focused on testing acute Aβ_42_ effects, but we do not exclude the possibility that the tau mutation may also play a role in altering the functional activity of mPFC pyramidal neurons at early-stage and/or later-stage AD.

To our knowledge, there is no published work of this type in the field to reveal the inability of Aβ_42_ in disturbing the normal functional activity of cortical pyramidal neurons in young healthy brain, and its capability in worsening cortical neuron dysfunction at the early stage of AD. Because 1) the earliest Aβ accumulation occurs in the orbital/mPFC and HIPP of elder patients at later stage AD (Palmqvist et al., 2017), 2) Aβ is preferentially released by dysfunctional pyramidal neurons and accumulated in the mPFC/HIPP (Cirrito et al., 2008), and 3) Aβ plaques form abnormal but functional L-channel-like Ca^2+^ channels to worsen the pyramidal neuron hyperactivity and [Ca^2+^]_in_ dyshomeostasis in the brain during the later stage of AD and aging (Arispe et al., 1993; Pollard et al., 1993; Abramov et al., 2004; Lal et al., 2007; Shirwany et al., 2007; Itkin et al., 2011), our findings suggest that Aβ is *not* a causative factor that triggers AD, but is one of the consequential effects of AD due to and following the dysfunction of pyramidal neurons in the mPFC/HIPP during the later stage of AD and aging (Figure 7).

In addition, the present study also indicates that there was no increase in Ca^2+^ influx via VGCCs in young mPFC pyramidal neurons in early-stage AD, likely due to the absence of Aβ plaques and lack of Aβ-formed Ca^2+^ channels, in such early stage of AD. Based upon this finding, we predict that the hyperactivity and overexpression of L-channels, including those formed by Aβ, will occur at the late stage of AD, which will interplay with overactive NMDARs to exacerbate [Ca^2+^]_in_ dyshomeostasis in pyramidal neurons, thereby inducing neurotoxicity (Figure 7), as we observed in another neurodegenerative disease (Napier et al., 2014; Khodr et al., 2016; Du et al., 2022).

Our perspective agrees with the reality seen in clinical practice. Cumulating evidence indicates that, although blockade of overactive NMDA receptors (by FDA-approved memantine) has modest effects in improving clinical conditions at early-stage AD, likely due to decreasing excessive Ca^2+^ influx via NMDARs in hyperactive pyramidal neurons, it fails to do so at moderate and late-stage AD (Hu, 2020). Given that neuronal Ca^2+^ homeostasis and neurotoxicity are jointly regulated by both NMDARs and L-channels, interfering only one of them would have very limited effects; and that could even be compensated by severer overactivation of the other one, including those formed by Aβ. Therefore, a novel therapeutic approach is desperately needed to aim on reducing overactivation of *both* NMDARs and L-channels in hyperactive pyramidal neurons. Encouragingly, our previous studies show that combined, but not individual, antagonism of NMDAR and L-channel overactivation is more effective than memantine or L-channel blocker alone to diminish hyperactivity, excessive [Ca^2+^]_in_, and neurotoxicity in pyramidal neurons (Napier et al., 2014; Khodr et al., 2016; Du et al., 2021; 2022) in the context of neuroHIV, another neurodegenerative disease that could also lead to dementia; and therefore, may be more efficient to interfere the progression of AD during aging (Figure 7).

Intriguingly, a recent report shows that long-term potentiation (LTP) is reduced in hippocampal pyramidal neurons in 3xTg-AD mice in later-stage AD with older age (Javonillo et al., 2022) (18 months; equivalent of ∼56-year-old humans). This result seems conflicting with our findings from the present study. However, it is worth noting that following persisting hyperactivity, the presynaptic storage of glutamate could be depleted; and therefore, its release from neurons’ terminals and dendrites is reduced, especially when responding to strong excitatory stimuli required for LTP. Furthermore, enduring increase of [glut]_o_ also causes desensitization and internalization of AMPARs and NMDARs into pyramidal neurons (Tong et al., 1995; Roche et al., 2001); both decrease neuronal responses to excitatory stimuli that previously induce LTP, and therefore could eliminate LTP.

Besides pyramidal neuron dysfunction in the brain, the present study also suggests astrocyte dysfunction that could exacerbate the dysfunction of cortical neurons. Cortical astrocytes regulate the normal activity of surrounding neurons. But that is disturbed by AD, and their dysfunction worsens hyperactivity and Ca^2+^ dyshomeostasis of neurons nearby, and *vice versa*. For example, increased glutamate release from hyperactive pyramidal neurons leads to abnormal elevation of [glut]_o_ in the mPFC and HIPP of AD brain. Aβ also inhibits glutamate uptake (Lauderback et al., 1999) and interrupts K+ homeostasis (Cheung et al., 2015; Price et al., 2021). These changes not only increase [glut]_o_ by inhibiting the activity of glutamate transporters in astrocytes, but also elevate [K^+^]_o_ by disrupting the astrocytic *K* ^*+*^ *spatial buffering* (Olsen and Sontheimer, 2008; Rimmele et al., 2017) and *K* ^*+*^ *siphoning* (Kofuji and Newman, 2004; Butt and Kalsi, 2006); both are mediated by K_ir_4.1 channels that are highly expressed in astrocytes, but not in neurons (Butt and Kalsi, 2006; Djukic et al., 2007; Seifert et al., 2009). An aberrant increase in [glut]_o_ and [K^+^]_o_ promotes the membrane depolarization of pyramidal neurons, potentiating their hyperactivity. Further, such astrocyte dysfunction in causing [glut]_o_ and [K^+^]_o_ dyshomeostasis also worsens each other (Figure 7). However, this unique astrocyte dysfunction is understudied in the field; and therefore, requires more attention and investigation in the future.

Aging is a key risk factor of AD, which gradually but substantially disturbs functional activity of cortical pyramidal neurons in the brain. Our previous study indicates that aging *alone* leads to a decline in the functional activity of mPFC pyramidal neurons (firing) and [Ca^2+^]_in_ dysregulation, which are mediated by dysfunction of L-channels and K^+^ channels (Khodr et al., 2018; Chen et al., 2019). Under these circumstances, Aβ plaques and Aβ-formed Ca^2+^ channels, which occurred during the late stage of AD and aging would exacerbate Ca^2+^ dyshomeostasis, and ultimately intensify neurotoxicity in the brain regions that regulate neurocognition. Additionally, investigation is also needed to define whether and how tau protein tangles alter the functional activity of cortical pyramidal neurons and astrocytes during AD progression.

While assessing the effects of early AD in male 3xTg-AD mice, it is critical to be mindful of the sex differences in neurochemistry, neuropathophysiology and behavior of 3xTg-AD mice, where female 3xTg-AD mice appear to be more vulnerable than male 3xTg-AD mice (but mainly at later-stage). The novel findings from the present study will help direct future studies focusing on female 3xTg-AD in early- and late-stage AD.

In summary, the present study demonstrates that pyramidal neurons in the mPFC become hyperactive in early-stage AD, in the absence of Aβ plaques and Aβ-formed Ca^2+^ channels. Such hyperactivity is mediated by overactive AMPARs and NMDARs. Naïve mPFC pyramidal neurons in young brain are insensitive to Aβ_42_. But Aβ_42_ worsens hyperactivity of cortical neurons in early-stage AD. Collectively, our novel findings suggest that Aβ is not a causative factor that triggers AD; rather, it is one of the consequential effects of cortical/hippocampal pyramidal neuron dysfunction, which, in turn, exacerbates neuronal dysfunction and [Ca^2+^]_in_ dyshomeostasis in the brain during the late stage of AD and aging. Additionally, our findings also suggest a dysfunction of the L-channel (including those formed by Aβ) during the late stage of AD and aging, along with a disrupted interaction between neurons and astrocytes in the brain. These novel findings could advance our understanding of the mechanism that underlies AD, which may also provide insights for developing novel therapeutic approaches.

## Data Availability

The original contributions presented in the study are included in the article/Supplementary Material, further inquiries can be directed to the corresponding author.

## Supplementary material

SUPPLEMENTARY FIGURE S1 Individual sEPSC events from the sampled non-Tg (*n* = 22 neurons/8 mice) 3xTg-AD (*n* = 19 neurons/7 mice) groups. **(A)** sEPSC event amplitudes from each non-Tg (black) and 3xTg-AD (red) neuron. **(B)** IEIs from each non-Tg (black) and 3xTg-AD (red) neuron.

SUPPLEMENTARY FIGURE S2 Individual sIPSC events from the sampled non-Tg (*n* = 14 neurons/6 mice) 3xTg-AD (*n* = 15 neurons/8 mice) groups. **(A)** sIPSC event amplitudes from each non-Tg (black) and 3xTg-AD (red) neuron. **(B)** IEIs from each non-Tg (black) and 3xTg-AD (red) neuron.
