# Supplementary figures and images for: Hyperactivity of medial prefrontal cortex pyramidal neurons occurs in a mouse model of early-stage Alzheimer’s disease without β-amyloid accumulation
Source: Front Pharmacol. 2023 Jul 3;14:1194869. doi: 10.3389/fphar.2023.1194869 (PMC10350500; doi:10.3389/fphar.2023.1194869)

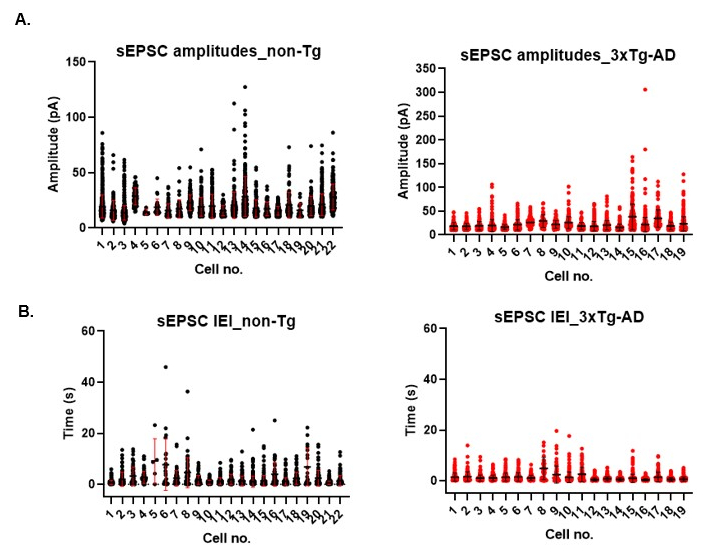

Supplement: Supplementary file 1 [file Image1.JPEG]

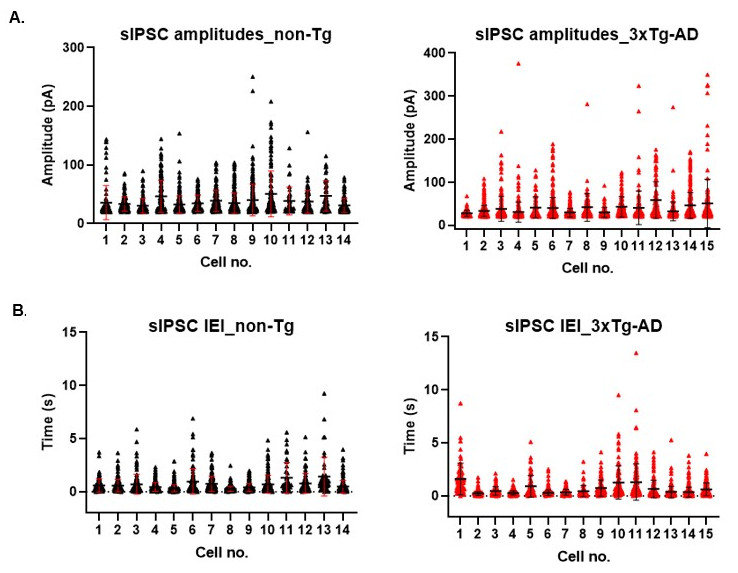

Supplement: Supplementary file 2 [file Image2.JPEG]
